# Underwater Application of Quantitative PCR on an Ocean Mooring

**DOI:** 10.1371/journal.pone.0022522

**Published:** 2011-08-01

**Authors:** Christina M. Preston, Adeline Harris, John P. Ryan, Brent Roman, Roman Marin, Scott Jensen, Cheri Everlove, James Birch, John M. Dzenitis, Douglas Pargett, Masao Adachi, Kendra Turk, Jonathon P. Zehr, Christopher A. Scholin

**Affiliations:** 1 Monterey Bay Aquarium Research Institute, Moss Landing, California, United States of America; 2 Lawrence Livermore National Laboratory, Livermore, California, United States of America; 3 Laboratory of Aquatic Environmental Science, Kochi University, Kochi, Japan; 4 Department of Ocean Sciences and Earth and Marine Sciences, University of California Santa Cruz, Santa Cruz, California, United States of America; Vanderbilt University Medical Center, United States of America

## Abstract

The Environmental Sample Processor (ESP) is a device that allows for the underwater, autonomous application of DNA and protein probe array technologies as a means to remotely identify and quantify, *in situ*, marine microorganisms and substances they produce. Here, we added functionality to the ESP through the development and incorporation of a module capable of solid-phase nucleic acid extraction and quantitative PCR (qPCR). Samples collected by the instrument were homogenized in a chaotropic buffer compatible with direct detection of ribosomal RNA (rRNA) and nucleic acid purification. From a single sample, both an rRNA community profile and select gene abundances were ascertained. To illustrate this functionality, we focused on bacterioplankton commonly found along the central coast of California and that are known to vary in accordance with different oceanic conditions. DNA probe arrays targeting rRNA revealed the presence of 16S rRNA indicative of marine crenarchaea, SAR11 and marine cyanobacteria; in parallel, qPCR was used to detect 16S rRNA genes from the former two groups and the large subunit RuBisCo gene (*rbcL*) from *Synecchococcus*. The PCR-enabled ESP was deployed on a coastal mooring in Monterey Bay for 28 days during the spring-summer upwelling season. The distributions of the targeted bacterioplankon groups were as expected, with the exception of an increase in abundance of marine crenarchaea in anomalous nitrate-rich, low-salinity waters. The unexpected co-occurrence demonstrated the utility of the ESP in detecting novel events relative to previously described distributions of particular bacterioplankton groups. The ESP can easily be configured to detect and enumerate genes and gene products from a wide range of organisms. This study demonstrated for the first time that gene abundances could be assessed autonomously, underwater in near real-time and referenced against prevailing chemical, physical and bulk biological conditions.

## Introduction

Modern molecular biological techniques have revolutionized our understanding of the diversity, function and community structure of marine microorganisms (for review see [1]–[3]). One of the techniques commonly employed in this regard is quantitative PCR (qPCR; [4]) which is used to detect and enumerate unique nucleic acid sequences indicative of specific organisms, functional genes and other genetic markers in samples collected from a wide array of environments [5]–[9]. For the vast majority of ocean science and resource management applications of qPCR, discrete samples are collected and preserved in the field, then returned to a laboratory where they are generally processed in batch mode many hours, days, or months later to reveal targets of interest. Rarely is qPCR applied in the field, adaptively, in support of ocean research and monitoring. When that capability is utilized, it is typically accomplished by setting up a temporary laboratory that embodies the essential elements of shore-based facilities (e.g., onboard an oceanographic research vessel). In either case, whether returning samples to a conventional laboratory or creating a portable laboratory that is used at sea, limited sampling opportunities often restrict our ability to document microbial community dynamics and fundamental biogeochemical transformations that microbes mediate in response to ephemeral environmental fluctuations [10]–[12]. Thus, opportunities to alter sample acquisition schemes or adapt experimental procedures based on results of qPCR assays are limited.

These restrictions have spurred the idea of developing “ecogenomic sensors” – a new class of autonomous sensor that enables the use of molecular biological techniques in an ocean observing framework [13]–[15]. We define an ecogenomic sensor as being a field deployable instrument that allows for hands-off sample collection, processing and molecular analytical analyses. The devices are meant to be completely autonomous and support two-way communications for transmitting results of analyses as well as for downloading instructions so that their mode of operation can be altered remotely [16].

A number of portable and/or reusable instrument systems that incorporate nucleic acid extraction and detection have been designed for use with environmental samples [13], [17]–[27]. However, most of the systems described are not suited for deployment in aquatic environments and often require some type of laboratory infrastructure or personnel to facilitate sample collection and processing.

Here, in a step towards realizing the ecogenomic sensor vision, we demonstrate for the first time the application of qPCR using the Environmental Sample Processor (ESP, [15]) on a coastal mooring. The instrument was used to remotely assess alterations of the microbial community structure and functional gene abundance in response to changing environmental conditions in Monterey Bay, California. Previous near real-time applications of the ESP utilized DNA and protein arrays to sense target molecules (rRNA or an algal toxin; [28]–[36]). The ESP has also shown its utility in collecting and returning samples for fluorescent in situ hybridization studies [29], [30], [32], [33] and transcriptomic analyses [37].

**Figure 1:**
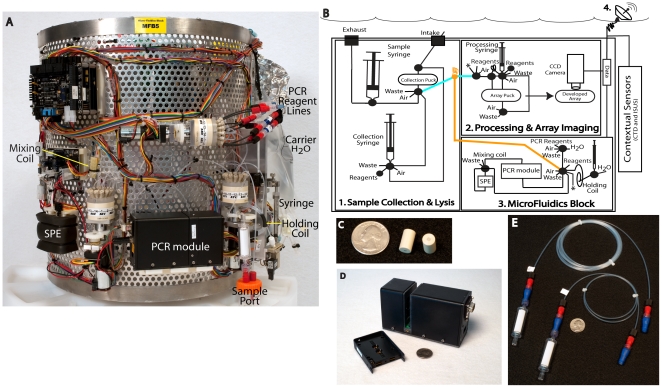
Photos of a standalone microfludic block (MFB, A), schematic representation of its incorporation into the ESP (B), and specific MFB components (C–E). A single fluidic connection linked the MFB and ESP (B, orange line) and permitted access to samples collected and partially processed by the ESP. The ESP sampled the environment via an intake valve and concentrated particulates in a collection puck containing a filter of the appropriate size (B-1). Cells were lysed in a chaotropic buffer and the lysate was positioned in a line (blue) between where the sample was collected and processed (B-2). Here, the lysate could proceed down two different paths. The processing syringe first delivered lysate to and began processing the DNA probe array for imaging. It then modified a second lyaste aliquot and positioned it in a port (orange) that is accessible to the MFB (B-3). The lysate then entered the MFB for nucleic acid extraction followed by serial qPCR. Additional ports of entry were available to the user on the ESP and MFB for loading user processed samples or standards (asterisks). Data from qPCR reactions, array images, and contextual data stored on the ESP were sent to a shore-side station via a surface radio buoy (B-4) hourly. The solid phase extraction column (C), qPCR module (D), and PCR reagent coils (E) are pictured.

The ESP was originally designed to concentrate particulate matter and either preserve it for subsequent laboratory analyses or homogenize it in preparation for molecular analytical tests that operate with sample and reagent volumes from 200 µL to milliliters. While this is appropriate for some detection technologies, others, like quantitative PCR (qPCR) demand precise µL scale fluid handling. To meet the latter need, we designed a separate fluid handling system that can be used as a stand-alone benchtop instrument, or be added to the ESP system for field deployment. We refer to this system as the “microfluidic block”, or MFB (Figure 1a).

In this contribution, we describe the development and application of methods that utilize a reusable solid phase nucleic acid extraction system and 2-channel real-time PCR module that was integrated with the MFB and ESP. The integrated system was deployed in the ocean for one month, demonstrating for the first time the ability to remotely track changes in microbial populations, *in situ*, using qPCR. This study highlights the potential of ecogenomic sensors and provides an example of a new way to study microbial communities in ocean observing networks.

## Materials and Methods

### System Overview

The core ESP is a robotic device that allows for underwater sample collection, DNA and protein probe array processing, and data transmission [15], [38]–[40]. A core ESP joined with the MFB (then called ESP-MFB) accomplished the same tasks, with the additional ability to perform DNA purification and qPCR.

Integrated use of the ESP-MFB (Figure 1b) began with opening a path between the external environment (ocean) and the collection stage to concentrate particulates. Sample collection and processing by the core instrument occurred in reaction chambers or “pucks” that were preloaded with appropriate filter media. For bacterioplankton studies, a sample of up to 1 L was collected onto a 25 mm diameter, 0.22 µm pore size hydrophilic Durapore filter (GV, Millipore) as described previously [33], [35]. The material retained on the filter was homogenized in 1.4 mL of 3 M guanidine thiocynate, pH 8.9 (3 M GuSCN, 50 mM Tris, 15 mM EDTA, 2*%* Sarkosyl and 0.2*%* SDS (v/v), at pH 8.9; modified from Tyrell et al [41], Saigene Corp.) at 85°C for 8 min. The resulting cell lysate was then filtered through a second puck containing a 0.22 µm pore size Durapore filter and transferred to the sample line between the processing and collection stages of the ESP (Figure 1b, blue line). The processing stage appropriately modified and distributed the lysate for downstream ribotype array and/or qPCR reactions. Once modified lysates were distributed, the collection stage of the instrument was available to initiate the next sampling event, typically one in which material was chemically preserved (“archived”) for analyses after the instrument was recovered. Processing of the ribotype array as well as all operations related to qPCR and sample archival continued in parallel.

For bacterioplankton ribotype arrays, 0.5 mL of lysate was combined with an equal volume of lysis diluent (50 mM Tris, 15 mM EDTA, 2*%* Sarkosyl and 0.2*%* SDS, at pH 8.9) then delivered to the probe array for hybridization. Methods for fabricating and developing DNA probe arrays for bacterioplankton clades are described elsewhere [30], [33], [35] except that biotynlated probes (139 ng/uL in 0.28 mg/µL strepavidin (Sigma), 497 mM NaCl, 3 mM EDTA, 100 mM Tris-HCl, pH 7.4) were printed on Optitran BAS83 (Whatman, [33]) using a non-contact microarray printer (Piezorray, PerkinElmer, Downers Grove, IL). Target rRNA abundances were estimated from the average probe spot intensity (n = 7–10 per DNA probe) using standard curves generated from reverse transcribed RNA of cloned 16S rRNA genes as described previously [35]. Raw hybridization values are presented for the marine cyanobacteria, as standard curves were not available for that assay at the time this work was done.

Once array hybridization began, a separate aliquot of the original sample lysate (250 µL) was conditioned for nucleic acid extraction by adding 225 µL SPE diluent (555 mM sodium acetate pH 5.2 in 70*%* ethanol (v/v)) in a mixing coil fitted to the processing stage of the core ESP. The modified lysate was then positioned into an injection loop valve (Figure 1b, orange circle); that valve was the common junction between the ESP's collection and processing stages in the core instrument and the MFB. Actuation of the injection loop valve then made modified lysate available to the MFB and maintained separate fluidic connectivity between collection and processing stages. At that point, the MFB initiated a sequence of protocols for nucleic acid purification and qPCR as described below.

Operation of the ESP-MFB was flexible. It could be configured to concentrate particles from a range of sample volumes and then parse a sample for both an array and qPCR analyses as outlined above, or direct sample homogenates to just one of those assays. The ESP-MFB can also be programmed to skip sampling and generate a “negative lysate”. The negative lysate was used to ascertain the cleanliness of the entire ESP-MFB system. In addition to the fully integrated path of seawater sampling, we also manually introduced partially processed samples (natural sample or control lysates) at different junctions within the system. These different entry points (Figure 1b, asterisks) were used to test the system and/or operate the MFB as a standalone unit. In addition to lysates, purified DNA templates were also introduced directly to a valve port on the MFB.

### The Microfluidic Block (MFB)

The MFB acted as an interface between the sample collection and homogenization functions provided by the core ESP and “downstream” operations associated with a solid phase extraction (SPE) column and qPCR module (Figure 1c and d). The MFB was based on concepts of sequential injection analysis [42] and zone fluidics [43]. It was operated as either a standalone unit or joined to the core ESP to permit fully autonomous analysis. Integration of the MFB and core ESP was accomplished through a single fluidic and electrical connection.

Fluidic movements on the MFB are controlled by a 1.0-mL syringe. The syringe is connected to carrier fluid (pure water, Sigma, St. Louis, MO) on one side and a 1.3-mL holding coil on the other (Figure 1a and b). The holding coil acts as a staging area to allow reagents to be accessed then delivered to another location in the MFB without entry into the syringe itself. The syringe, in combination with four valves, permits access to the injection loop coil on the core ESP, sample ports, reagents, carrier fluid, clean-air, waste containers, the solid phase extraction (SPE) column, and qPCR module. All lines on the MFB are PFA 1/32 inch tubing except for the line through the qPCR module that consisted of FEP tubing (0.04 inch ID×0.0625 inch OD, Medical Extrusion Technologies, TX).

#### MFB Nucleic Acid Extraction

Nucleic acids were extracted from lysate provided from the injection loop coil on the core ESP or supplied by the user on a sample port. All fluidic movements for nucleic acid extraction occurred at 5 µL/sec except for elution of template which was performed at 1 µL/sec. Four-hundred µL of lysate was pushed to waste through a custom-made silica-packed 10×2 mm HPLC column with 2 µm pore size titanium frits (Figure 1c, Orochem Technologies, Lombord, IL) held at 55°C in a temperature-controlled insulated aluminum block. After delivery of the lysate through the column, the line that delivered the lysate was rinsed with water and then cleared with clean air. The column was then rinsed with 0.330 mL column wash buffer (100 mM NaCl, 10 mM Tris-HCl pH 7.8, 2 mM EDTA pH 8.0, in 70*%* (v/v) ethanol) followed by 1.45 mL of air. Nucleic acids were eluted from the column with 60 µL of water (Sigma) into a serpentine mixing coil (Global FIA, Fox Island, WA) to obtain a well-mixed extract. To aid in column decontamination, 0.1 mL of 20*%* bleach (Clorox, Oakland, CA) was positioned across the column, incubated at 95°C for 2 min, and remained in the column until full decontamination of the MFB had begun. Extracted nucleic acids held in the serpentine mixing coil were then primed to the valve connected to the 1.3-mL holding coil and could either be recovered for bench analysis, used for qPCR reactions on the MFB, or both.

To test the efficiency of nucleic acid extraction by the MFB, we compared MFB nucleic acid extractions originating from DNA-spiked lysates and natural samples with those using a modified DNeasy protocol (Qiagen, Valencia, CA). For the spiked DNA lysate tests, a known concentration of calf thymus DNA (Sigma) was added to the lysis buffer then modified with SPEdiluent in the ratio described above. A portion of the lysate was loaded onto one of the sample ports on the MFB and a second equal portion extracted using a DNeasy column (see below). Nucleic acid extraction on the MFB proceeded as described. At the end of the procedure, the eluted DNA was then recovered in total or in 10 ul aliquots from the sample port. Natural samples were processed similarly, but were lysed in 1.4 mL of lysis buffer at 85°C for 10 minutes, filtered through a 0.2 µm syringe filter (Millipore), then modified as above. The eluted nucleic acids recovered from the MFB were compared to the bench extracted material spectrophotmetrically (Nanodrop, Wilmington, DE) and/or by qPCR using an ABI7700 (Applied Biosystems, Carlsbad, CA).

DNA extraction using a modified DNeasy protocol occurred as follows. An equal portion of the modified lysate (0.4 mL) extracted on the MFB, was passed through a DNeasy column. The DNeasy column was then rinsed and dried according to the manufacture's suggested protocol (Qiagen). Nucleic acids were eluted in 60 µL with AE buffer.

Between sample extractions on the MFB, lines for SPE were decontaminated. First, the residual template was pushed to waste. The path for SPE was then flushed with 20*%* bleach, rinsed with water, and the water displaced with clean air. The order of decontamination was as follows: bleach was pushed into the serpentine mixing coil, then the sample port and injection loop flushed, and lastly the path across the column in both directions. Finally, bleach was removed from the serpentine mixing coil, rinsed with water and left dry.

#### MFB PCR Module

The custom made, two-channel PCR module developed at the Lawrence Livermore National Laboratory (Figure 1d) is a small, low-power, flow-through device that accommodates user-defined temperature cycling parameters and optical integration time for reading reporter dye emissions [18], [22], [27]. The LED light sources and detectors were compatible with assays utilizing FAM, SYBR green, TAMARA, and NED or other dyes with similar excitation/emission spectra. Assays utilizing SYBR green can be further analyzed by dissociation curve profile; this functionality was not employed for the present study.

The valve arrangement holding the qPCR reagents permitted sequential mixing and thermocycling of up to 6 qPCR assays per template purified using the MFB's SPE system. Reagents for qPCR assays consisted of two separately stored reagents: an enzyme mix containing MgCl_2_ if necessary and the primers with the 5′ nuclease probe.

The MFB automatically performs all steps required to mix and thermally cycle a qPCR reaction. qPCR reactions (30 µL final volume) were assembled by combining 18 µL of enzyme mix with 6 µL of primer/probe solution, and isolating that mixture between two, 6 µL bubbles of air. This mixture was positioned mid-way across a valve port where 6 µL of sample template was added. Selection of the template was varied depending on requirements: water for a no template control (NTC), eluates from the SPE column (natural samples or “negative lysate”), or user-primed DNA standards (positive controls). The complete reaction mixture was then positioned within the PCR module where it was isolated fluidically and thermocycled with the desired conditions (see below). All fluidic moves for assembling and positioning a PCR reaction were performed at 1 µL/sec.

At the completion of each PCR reaction, the lines for qPCR were decontaminated with 20% bleach then rinsed with water [22]. To aid in elimination of amplicons in the PCR tubing, the line within the module was held at 95°C for 10 minutes with 20*%* bleach then rinsed with water. A 45-cycle qPCR reaction followed by decontamination took 2 hours to complete and consumed 14.2 Wh.

Resulting data was plotted as the change in fluorescence of the reaction (raw fluorescence of each cycle minus the background fluorescence) versus cycle number [4]. Background fluorescence of the reaction was determined during the early stages of PCR, before target amplification was detectable by the instrument. An appropriate cycle threshold (Ct) was determined using reactions from the NTC and positive control plasmids. Once an appropriate cycle threshold (Ct) was determined for a given assay, it was held constant for an entire reagent load.

In order to derive semi-quantitative information on gene abundances in natural samples, standard curves for each qPCR assay were run serially from highest to lowest dilution in duplicate or triplicate. Standards for each assay were made from serial dilutions of linearized plasmids (10^2^–10^5^ target copies) in nuclease free water. For use on the MFB, an excess volume of the standard dilution was primed to the sample port and multiple reactions in series were run for a single dilution. After a standard dilution series was completed and before running the next dilution, the sample port delivering the template was decontaminated with 20*%* bleach and rinsed with pure water.

To test sources of sample carryover in the system, reproducibility, and effectiveness of the decontamination, a series of tests involving manually made lysates from replicate field samples, NTCs and negative lysates were processed in series. The extracts were analyzed by the qPCR module on the MFB using the SAR11 16S rRNA gene assay. A series of five qPCR reactions were run on each field-collected sample. Between replicate field samples, one NTC reaction and one reaction from a negative lysate were performed. Results obtained using the MFB were compared to the same sample lysates extracted using the modified DNeasy protocol and analyzed using the ABI7700.

If a lysate was provided to the core MFB by the ESP, then the entire fluid path in the ESP from the sample intake to the processing syringe was cleaned with 20% bleach and rinsed with flush (0.5*%* Tween20 (v/v), Sigma). Afterwards, the intake line was filled with 0.2*%* hypochlorite (v/v, Sigma) until the next sample event when it was displaced with flush. Upon completing the last qPCR reaction for a given sample, the path for solid phase extraction was decontaminated followed by that for qPCR as described above. At that point, a new ESP-MFB sampling event involving qPCR analysis could be initiated.

### Reagents

The ESP-MFB carried all the reagents necessary to fully process a sample. Spent reagents were collected into internal waste containers. All reagents except those for qPCR were sterilely transferred or 0.2 µm filtered (Sterevix GV, Millipore) into Flexboy bags (Sartorius-Stedim, Bohemia, NY). All reagents were primed directly to the appropriate reagent valve port, except for those on the MFB that contained bleach or ethanol. For the latter, a 10 µL air bubble was positioned between the reagent and valve port between use of those fluids to minimize their interaction with samples as the valve rotor moved.

Real-time qPCR assays included a commercially available internal positive control (IPC, Applied Biosystems) to verify instrument functionality and reagent stability, as well as the16S rRNA genes of SAR11 [6] and marine crenarchaea [5], and the large subunit gene of ribulose-1, 5 bisphosphate carboxylase oxygenase (RuBisCO, *rbcL*) from abundant *Synechococcus* clades in Monterey Bay [44]. Primers and probe sequences and concentrations in reactions for genes found in the environment are presented in Table 1. Reaction conditions for 5′ nuclease analysis were as follows: all reactions (30 µL total) contained 1× AccuPrime Supermix I (Life Technologies) with the addition of 2.5 mM MgCl_2_. The IPC assay contained 0.5× of a custom NED/MGB-labeled primers-probe solution and 2.5× template (TaqMan Exogenous Internal Positive Control, Part No. 4308323). Hydrolysis probes for environmental targets were labeled with FAM/BHQ-1. The thermocycling profile on the MFB's PCR module for all assays was 90°C for 75 seconds, followed by 42 cycles of 59°C for 30 seconds with diode reading, 72°C for 15 seconds, and 90°C for 15 seconds. Laboratory reactions were performed as described above, but thermocycled in ABI7700 (Applied Biosystems) with an initial denaturation at 95°C for 75 seconds, followed by 40 cycles of 95°C for 15 seconds followed by 59°C for 60 seconds.

**Table 1:** Primers, 5′ nuclease probes, and characteristics of the qPCR assays run on the MFB.

		SAR11 16S rRNA gene	Marine crenarchaeal 16S rRNA gene	*Synechococcus rbcL*
qPCR primers and probes∧	Forward Primer	SAR11-433f CTCTTTCGTCGGGGAAGAAA (500 nM)	ARCHG1-334F AGATGGGTACTGAGACACGGAC (1000 nM)	RbcLfCAGACCACCCTCGGCTACAT (333 nM)
	Reverse Primer	SAR11-588R CCACCTACGWGCTCTTAAGC (1500 nM)	ARCHG1-554R CTGTAGGCCCAATAATCATCCT (500 nM)	RbcLrCCCAGTCCTGATCGAAGAAGTT (333 nM)
	5′ Nuclease Probe	TM519bR TTACCGCGGCTGCTGGCAC (200 nM)	TM519aRTTACCGCGGCGGCTGGCAC (400 nM)	TMrbcLTTCGTTCCTGAAGATCGCAGCCG (200 nM)
	Assay Reference	[6]	[5]	[44]
MFB	Δ fluorescence*	1000	1400	500
	NTC	No Amplification	No Amplification	No Amplification
	Slope	−3.8646	−3.5654	−3.1259
	Intercept	40.269	41.495	41.862
	R^2^	0.9949	0.9872	0.9473
	PCR efficiency	0.81	0.91	1.09
ABI7700	PCR efficiency	0.97	0.98	0.97

∧ final concentration in 30 µL reaction.

*at the end of a qPCR reaction run with the 10^4^ standard dilution.

PCR efficiency = 10^(−1/slope)^−1.

On the MFB, the enzyme and primers-probe solutions were contained in separate bleach-cleaned, coiled tubing with Microclave connectors (ICU Medical Inc., San Clemente, CA, Figure 1e). One end of the coil was opened to the atmosphere through a 0.2 µm Stervix filter (Millipore). Each reagent coil contained between 30 to160 reactions depending on the mission and included enough reagents to generate standard curves and accomplish a sequence of runs while deployed. Once loaded, all reagents were maintained in the dark (wrapped in foil) and held at ambient temperature (9–25°C).

### Sample Collection

Surface seawater samples used to test the system were collected from the Monterey Bay (Monterey Commercial Wharf and Station M0) by bucket. Seawater (0.5 to 1 L) was either processed by the ESP or vacuum filtered at <10 psi onto 0.22 µm GV filters (Millipore, Bedford, MA). Manually collected filters were either used immediately or flash frozen in liquid nitrogen and stored in liquid nitrogen or at −80°C until use.

### Field Testing

**Figure 2:**
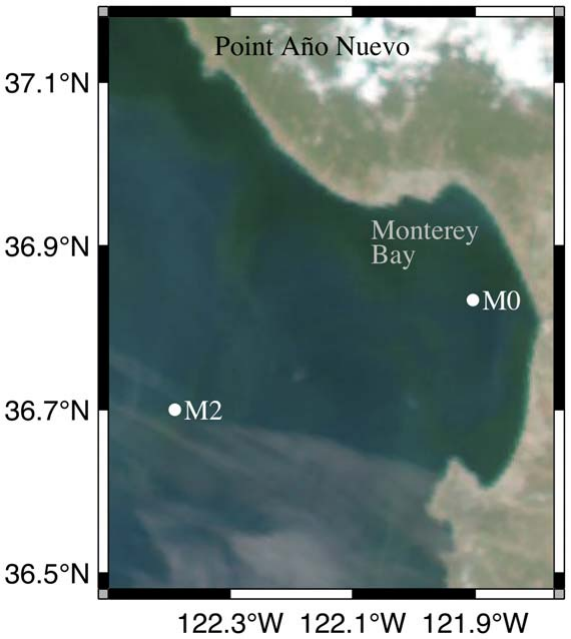
MODIS image from June 6, 2009 showing the locations of Station M0 in Monterey Bay, CA where the ESP was deployed and Station M2.

The ESP was deployed under permit number MBNMS-2005-010-A2 in Monterey Bay, CA at Station M0 (36.83N, 121.90W, Figure 2) from May 14 to June 11, 2009, on a mooring that maintains the instrument subsurface [45]. The ESP was deployed with additional environmental sensors including a Seabird SBE 16+CTD (Bellevue, WA) with fluorometer (Turner Cyclops-7) and transmissometer (WetLABS Cstar), and an In Situ Ultraviolet Spectrophotmeter (ISUS: [46]) for nitrate determinations. Measurements were taken every 12 minutes. In addition to environmental sensors on the ESP mooring, a Dorado class autonomous underwater vehicle (AUV; [47]) was tasked with surveying a volume of water surrounding the ESP in order to resolve the nature of water mass changes at the mooring. During June 1–4, the AUV repeatedly (15 times) mapped a volume ∼2 km×2 km in horizontal extent over the upper 25 meters. Wind direction and speed at the M2 mooring (36.70 N, 122.39 W; Figure 2) was used to characterize the regional wind forcing that influences water mass changes in the bay (e.g. see [36]).

The ESP collected samples throughout the deployment for *in situ* near real-time analysis as well as to archive material for metatranscriptomic analyses; results associated with the latter are presented elsewhere [37]. Real-time analyses of each sample included a ribotype array and a sequence of qPCR assays run serially in the following order: the IPC, the 16S rRNA gene from SAR11, the 16S rRNA gene from group 1 marine crenarchaea, and the large subunit ruBisCO (*rbcL*) from *Synechococcus* (Table 1). Prior to deployment, reagents for 80 reactions per assay were loaded on the MFB.

During field operations, the ESP collected and processed 22 field samples using both ribotype arrays and qPCR as described above. In addition, two negative control runs (negative lysate) were interspersed among the native samples to assess system-wide contamination. NTCs were run following the negative lysate to specifically assess PCR reagent and module contamination. Array images, results of qPCR runs, a log of instrument operations and data from the CTD and ISUS were transmitted to shore hourly by radio modem.

## Results

Modification of the ESP to accommodate qPCR required development of a fluid handling system (the micro-fluidic block; MFB), a reusable solid phase extraction (SPE) column for DNA purification, and a flow-thru, real-time PCR module (Figure 1). Initially, nucleic acid extraction and qPCR on the MFB was demonstrated in a standalone mode and then the MFB was attached to the ESP to provide an integrated, fully autonomous system capable of live sample acquisition to reporting of results while deployed below the ocean surface.

### Nucleic acid extraction by the MFB

In general, the extraction efficiency of the SPE column was similar to or better than that of the DNeasy column regardless of what port was used to introduce the sample to the MFB (Figure 1b, asterisks), albeit with a wider variance depending on the particular SPE column used (n = 10; 88–160% of DNeasy). We also found that with repeated use, nucleic acid recovery from a single column using the protocol described here decreased approximately 25% after 30 extractions (approximately the same number of samples that would be collected during a 1 month ESP deployment).

Of the 60 µL of water used to elute DNA from the SPE column, approximately 55 µL was recovered. After priming to a valve prior to assembling PCR reactions, this volume was sufficient to provide 6 ul of template to six PCR reactions from any one of a number of enzyme mix and primer/probe combinations. To test the uniformity of the eluted DNA, five10 µL aliquots from a single extraction were recovered and examined spectroscopically; the DNA concentration of those samples varied by 4.5%.

MFB extraction methods had no effect on PCR amplification (ABI 7700) using natural samples. A comparison of four 10 µL aliquots of a nucleic acid extract recovered from the MFB and the single eluate from the DNeasy column produced similarly shaped curves (data not shown) and the Ct's observed were 20.2±0.2 (n = 4) and 19.8, respectively. Thus, although the efficiency of the SPE column declined with repeated use, the extract produced was well mixed and comparable to those produced using conventional methods.

### qPCR Assays

**Figure 3:**
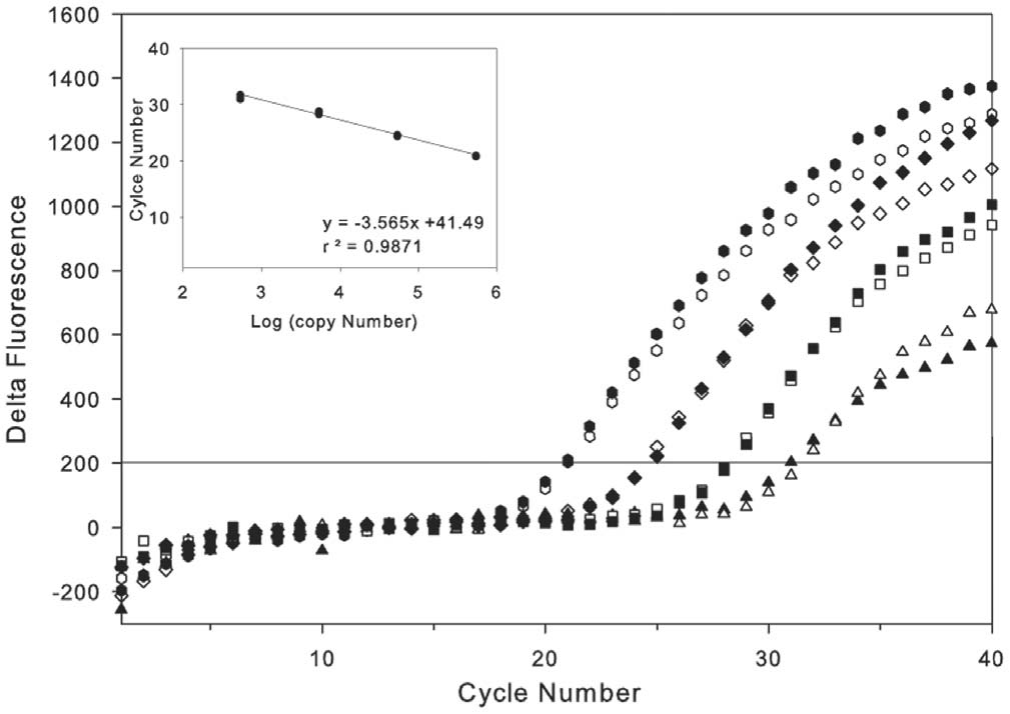
Example of background subtracted standard reactions from ten-fold dilutions of a linearized plasmid containing the 16S rRNA gene from marine crenarchaea. The inset shows the conversion from Ct to copy number of 16S rRNA genes. See table 1 for assay details. Symbols from lowest to highest dilution are circles (5.4×10^2^ copies/reaction), diamonds (5.4×10^3^ copies/reaction), squares (5.4×10^4^ copies/reaction) and triangles (5.4×10^5^ copies/reaction), respectively. Replicates from one dilution are the same symbol (open and closed).

**Figure 4:**
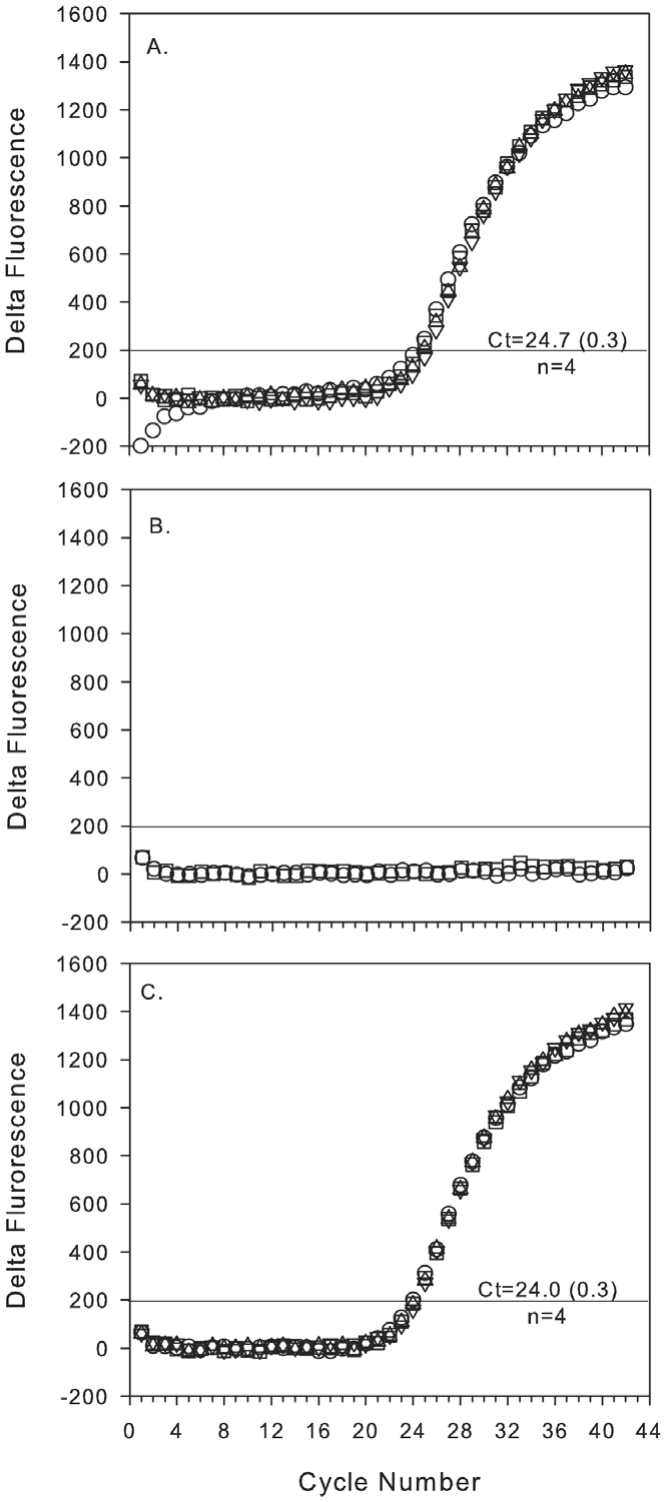
Reproducibility and performance of the decontamination protocols between replicate field samples analyzed by the MFB. Panels show amplification curves of SAR11 16S rRNA genes from samples processed in the following order, field-collected sample (A), NTC (B, squares), a negative lysate (B, circles) and a replicate field sample (C). The above series was repeated a total of three times, only the first series is depicted. The average Ct (at delta fluorescence of 200; horizontal line) and standard deviation of the replicate reactions from one nucleic acid extract is shown for each field sample.

Comparisons of qPCR reactions built and thermocycled using the qPCR module on the MFB versus those made manually and analyzed using the ABI7700 revealed a consistent Ct shift between the MFB and commercial machine, regardless of template source. The shift was characteristic of the particular PCR module used and not a result of the MFB extraction procedure or poor reagent mixing (data not shown). Despite this difference, the PCR module was internally consistent when analyzing replicate templates (e.g. Figures 3 and 4) and provided similar estimates of target gene abundance based on relevant standard curves.

Standard curves for each assay were derived from replicate analyses of 10^2^–10^5^ copies per reaction (Figure 3, Table 1). Amplification below the 10^2^ standard was considered detectable, but unreliable, and therefore unquantifiable. Above 10^6^ copies per reaction there was a risk of contaminating the MFB with template that can only be eliminated by extensive cleaning protocols. Reaction efficiencies observed using the MFB were within an acceptable range (ABI publication note 136AP01-01) except for the SAR11 assay that was slightly below what was desired (Table 1). Since the primary objective of this study was to prove overall feasibility of using qPCR on a coastal mooring, no further attempt was made to optimize PCR conditions for use on the MFB.

In addition to standard curves, homogenates from field samples were prepared manually and delivered to the MFB for SPE and qPCR. Based on similarity of the amplification curves and Cts (e.g., Figure 4a), nucleic acids extracted by the MFB and made available for subsequent qPCR reactions were stable for at least 10 hours. By running a NTC with elution water as template we assessed sample-to-sample carry-over/contamination within the valves and tubing associated with operations related to assembling and thermocycling a PCR reaction (Figure 4b, squares). The absence of amplification indicated that the target previously found to be abundant in the field sample did not remain in the PCR system. Next, a negative lysate was processed and analyzed by the MFB (Figure 4b, circles) to determine the effectiveness of cleaning the entire SPE system. Again, no amplification was detected indicating that the system-wide cleaning procedure was sufficient for eliminating a relatively abundant target in a natural sample. The above process was repeated an additional two times with replicate field samples with the same results, proving the effectiveness of the decontamination process and reproducibility of qPCR within and between replicate extractions of a field sample (average Ct 24.2±0.5, n = 3 samples, 12 qPCR reactions [Figure 4, a and c]). Subsequent testing revealed that the column decontamination protocol only effectively reduces the Ct by 10–12 cycles. While SPE column decontamination was thus not 100% effective, less than 1% of the total copies of target from a previous sample remained in the column prior to processing the next sample. Consequently, for the purposes of this concept validation study, we interpreted qPCR data to indicate relative changes in abundances of the target groups over time rather than an absolute measure of gene abundances.

The IPC was used to monitor qPCR reagent stability and sample inhibition. The IPC run included the extracted template at the same concentration as that used for other assays as well as its target pre-mixed into the primer-probe reagent. In this study, the IPC reaction was run independent of and immediately prior to other assays targeting specific environmental genes. When run with elution water only (NTC), the IPC generated an average Ct of 34.4±0.9 (n = 27). The IPC from 59 of 64 seawater samples (biomass equivalent of ≤150 mL seawater was passed through the column) was within 1 standard deviation of the NTC. The remaining five runs either showed an increase in the Ct (n = 3) or failed to amplify (n = 2). Based on this experience, we limited sample volume applied to the SPE column to no more than the equivalent of material extracted from 150 mL of native water. The IPC was found to be very useful for ensuring the system was working (e.g., via NTC), and did on occasion reveal gross sample inhibition during laboratory trials, but it nevertheless could not be used to correct the Cts for other assays.

### Reagent stability

qPCR reagents protected from light and stored in tubing coils (Fig. 1e) on the MFB at room temperature showed remarkable stability for at least 2 months (Table 2). Oddly, we observed rapid degradation of the SAR11 and marine crenarcheal 16S rRNA gene and *rbcL* assays within one week following recovery of the instrument from its pressure housing post-deployment. This only occurred after a complete cycle of placing the ESP in its housing, deploying it, and recovering the instrument from the housing; reagents were stable while the instrument was in its pressure housing. Subsequent testing of the recovered reagents showed the FAM-BHQ primer probe mixes were compromised, but not the enzyme or IPC (data not shown). These results differed from FAM-BHQ stability tests run on the MFB while it was maintained in the laboratory. The mechanism underlying destabilization of the FAM-BHQ-labeled probe as result of cycling the instrument through the process of placing it in, and recovering it from the pressure housing is not yet understood.

**Table 2:** Stability of qPCR assays held at ambient temperature on the MFB.

Assay	Label	Test Length	Initial Ct	Final Ct
IPC	NED/MGB	5 months	34.2	33.2
Marine Crenarchaea	FAM/BHQ	4 months	27.0	26.9
SAR11	FAM/BHQ	2 months	28.8	28.6

### Field Testing of ESP-MFB

**Figure 5:**
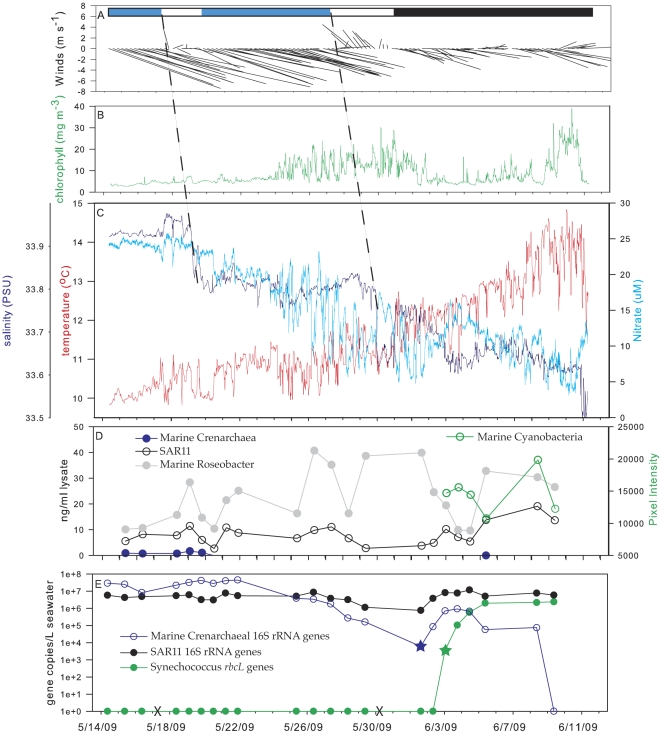
Environmental conditions and real time results from the ESP deployed at station M0, Monterey Bay from May 14–June 11, 2009. Hourly winds measured at station M2 that indicate regional forcing (A). Colored bar indicates strong upwelling conditions (blue), relaxation-reversal of upwelling favorable winds (white), and conditions dominated by local physical processes (black). The dashed lines show the lag response between the atmospheric and oceanographic data. CTD data (binned to 3 hours) from the moored ESP included chlorophyll (B), salinity, nitrate and temperature (C). Only a subset of the bacterioplankton groups detected on the ribotype arrays are shown (D). Array signals for SAR11, marine crenarchaea, and marine *Roseobacter* were converted to ng target rRNA per mL lysate using standard curves [35]. The average raw pixel intensity from array probe spots is presented for marine cyanobacteria. Marine crenarchaeal 16S rRNA, SAR11 16S rRNA and *Synechococcus rbcL* genes, expressed as copies per L seawater (E). Starred data points in panel E represent genes detected but unquantifiable (Ct<10^2^ standard) and Xs mark dates when the negative controls were run.

**Figure 6:**
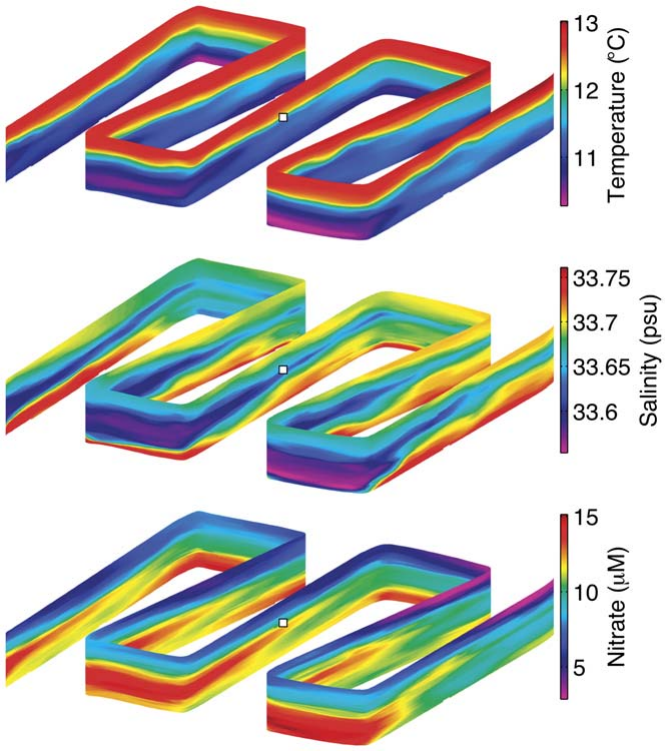
Water mass variability in temperature, salinity and nitrate concentration around the ESP mooring as mapped by an autonomous underwater vehicle (AUV). The synoptic survey was comprised of 138 profiles acquired in 3 hours, between June 2 22∶30 and June 3 01∶30, 2009. The survey domain is 2.3 km E-W and N-S and depth range 0–30 m. The view is from the NE. White box indicates the depth and position of the ESP during sampling.

The ESP was moored in Monterey Bay from May 14–June 11, 2009 where it ran 26 pre-scheduled assays that included 22 native samples processed using ribotype arrays along with two negative control (i.e., negative lysate and NTC) qPCR runs. Observed population shifts in the microbial community were clearly associated with varying oceanographic conditions (e.g., upwelling events and phytoplankton blooms, Figures 5 and 6). In addition, the Cts of the IPC assay for all the controls and native samples were within one standard deviation (average = 35.3±0.93), indicating no overt PCR inhibition.

Negative controls were run on the instrument pre-deployment, twice during the deployment (May 17 and May 30) and once post-deployment. For the assays targeting microorganisms from the environment, no amplification was observed for the NTCs or from the eluate of the negative lysates if the Ct of the previous field sample was >26. Residual template was detected in the eluate of all the negative lysates for SAR11, but only for the first negative control run in the field for marine crenarchaea. In each case, these groups were very abundant in the previous field sample (Figure 5). As expected, the Ct of the negative lysate was shifted 10–12 cycles higher than the preceding native sample. Thus, we estimate that system-wide sample carryover accounted for <0.3% of targeted genes to the subsequent native sample.

Regional winds were predominantly upwelling favorable (alongshore equatorward) during the first half of the deployment (Figure 5a, May 14–28). Consistent with this wind forcing, the presence of recently upwelled waters at the ESP mooring was indicated by relatively cold, saline and nitrate-rich conditions (Figure 5C). This pattern was interrupted by a wind relaxation during May 18–20 (Figure 5A), and oceanographic response to the wind relaxation was evident as a sudden decrease in salinity at the mooring (Figure 5C). These two periods, May 14–17 and May 20–28, were associated with different water mass characteristics and microbial communities. During the earlier period, marine crenarchaea were detected on the arrays and were found in high abundance as determined by qPCR. After May 20^th^, however, the marine crenarchaea were only detectable with qPCR (Figure 5e) except for the array on June 5^th^.

A significant relaxation-reversal of the upwelling favorable winds occurred during May 27–30, and thereafter upwelling winds became weaker and more intermittent than they were during the first half of the deployment (Figure 5a). This change in regional wind forcing was associated with warming of ∼3°C and freshening of ∼0.3 psu at the mooring during the second half of the experiment (Figure 5). Within this overall trend of warming and freshening were two periods of accelerated, inverse variation in salinity and nitrate, during approximately May 30–31 and June 2–5 (Figure 5C). Elevated nitrate in low salinity waters was unexpected for two reasons: (1) elevated nitrate concentrations are typically associated with relatively saline, recently upwelled waters, and (2) there was no apparent source of terrestrial freshwater supply to the mooring site. The first of these anomalous events was not sampled by AUV or ESP, however, the second event was sampled by both. AUV surveys revealed the movement of a cold, low-salinity, nitrate-enriched layer across the mooring in early June (Figure 6).

Concurrent with the anomalous chemical conditions during June 2–5 was a 100-fold increase in marine crenarchaeal abundance as detected by qPCR. By the end of the deployment, those organisms were below the detection limit of the assay. There was an overall positive correlation between nitrate concentration and the marine crenarchaeal abundance (y = 8.524.2e^0.4276x^ r^2^ = 0.7811). Also during this period, *rbcL* genes of *Synecohcoccus* were first detected with qPCR and then followed by concurrent signal from the ribotype arrays originating from marine cyanobacteria. Subsequent samples indicated a bloom of *Synechococcus* maximally reaching 7.2×10^6^ genes per L seawater on June 9^th^. In contrast, the presence of SAR11 rRNA and rRNA genes over the course of the whole deployment remained relatively constant regardless of the environmental conditions. Results regarding the distribution on these groups were as expected given the environmental conditions at the time of sampling, except for the detection of marine crenarchaea in the anomalous water layer that flowed across the mooring in early June.

## Discussion

The application of qPCR for assessing microbial genomic capacity and activity has provided a wealth of insights into understanding the distribution and function of organisms in response to environmental fluctuations [6], [7], [36], [48], [49], [50]. The goal of this study was to assess the feasibility of using that technique in a remote sensory context to advance both the concept and technology underpinning “ecogenomic sensors” [16]. Towards that end, we incorporated a real-time PCR instrument [18] within the ESP system. This required devising sample handling consistent with nucleic acid purification and qPCR protocols that allow the hands-off operation of re-usable flow-through system components, including a solid phase extraction column and PCR module. We tested reagent stability and the performance of the system with published assays of genes (rRNA or functional) from three abundant microbial groups associated with different oceanic regimes in Monterey Bay. This study demonstrated for the first time that gene abundances could be assessed autonomously underwater in near real-time and referenced against prevailing chemical, physical and bulk biological conditions (Figure 5).

### Sample handling and solid phase extraction

Sample collection and homogenization used here was applicable for both qPCR and sandwich hybridization methodology. This allowed us to leverage already developed methods for the collection and homogenization of samples for bacterioplankton community rRNA analysis [35]. A single lysate generated by the ESP could be modified for downstream processing of nucleic acid extraction and DNA probe arrays. Thus, each sample was analyzed by multiple detection methods, providing the ability to corroborate results obtained on the instrument.

Our method of nucleic acid extraction used on the MFB produced DNA of comparable quality and quantity to laboratory-processed samples. Matched samples had comparable extraction efficiencies and gene abundances as determined spectrophotometrically and by qPCR analysis, respectively. In addition, and as discussed in greater detail below, tests with integrated sample preparation and qPCR show that the SPE column is reusable, permitting serial processing and analysis of field samples after system decontamination.

### PCR reagent storage

Our method of reagent handing and storage was adequate for both laboratory testing and field deployments. All qPCR reagents were stored on the MFB at ambient temperature in coiled tubing for up to 5 months. This reagent storage method, although unconventional compared to normal laboratory procedures, showed little variability in assay performance for extended periods of time at ambient temperatures (Table 1). Similar reagent handling practices were used on the Autonomous Pathogen Detection System (APDS), however in that system the reagents were replenished weekly [18], [22], [27]. Long-term stability tests of reagents are critical, as subsurface operation of an instrument makes reagent re-supply difficult, if not impossible.

### Re-usable flow-through PCR module

Modifications to the flow-through PCR module originally developed for the APDS [18], [22], [27] were performed to meet specific requirements of the ESP. These included low power (10–14 volts), two-channel optical detectors, and interoperability with ESP control and data acquisition. The flow-through module performs sequential analysis of a template using a variety of primer-probe and enzyme combinations. Potential template sources included negative controls (no template and no sample controls), nucleic acids from field-collected samples or user-introduced standards.

We observed good reproducibility and PCR efficiency of reactions built and thermocycled on the MFB. Replicate runs of standards (Figure 3) and nucleic acids from field samples (Figure 4) produced similar Cts. Standard curves produced similar PCR efficiencies to those run on laboratory equipment (Table 1).

Both the SPE and qPCR module were reusable, flow-through devices so the components were cleaned with bleach and water between discrete sample processing events. These procedures resulted in no observed carryover of amplicons or templates between running standard reactions and no template controls. However, we did observe some carryover between environmental samples (<1%) and a loss in column extraction efficiency with repeated use (n = 30, 25% change). Both carryover and loss of extraction efficiency introduced negligible effects on the estimated gene abundances in field samples processed by the MFB. In fact, our procedures produced qPCR results that were reproducible within and between samples (Figure 4) and similar to laboratory-processed samples. Because of the issues discussed above and known bias inherent with nucleic acid extraction and qPCR [51], [52], we interpreted data from field samples detected with environmentally-targeted assays as a reflection of relative changes in microbial abundance, not a measure of absolute abundance.

At this point, we chose not to further optimize assay conditions. Instead, we focused on field trials to address the larger issues of operating autonomously, subsurface in the ocean. Those challenges included end-to-end, sample acquisition, preparation and processing for analyte detection coupled with data transfer to a shore side receiver.

### Field testing

For deployments of the ESP-MFB, we targeted genes of selected bacterioplankton groups known to be abundant in the different oceanic regimes in Monterey Bay. The historical time series investigations allowed interpretation and verification of results produced by the ESP. In addition, a commercially available internal positive control (IPC) was run on each sample to assess non-specific sample inhibition, provide confidence in negative results obtained from environmentally targeted assays, and verify the qPCR enzyme was not compromised. With the volumes of seawater processed by the ESP during the deployment, none of the field samples used in qPCR reactions exhibited inhibition.

While deployed, the ESP targeted members of the bacterioplankton community in seawater samples using ribotype arrays and qPCR assays for the 16S rRNA gene of SAR11, the 16S rRNA gene of marine crenarchaea, and the large subunit RuBisCO (*rbcL*) of *Synechococcus*. In addition, sensors on the ESP mooring, other Monterey Bay moorings, and an autonomous underwater vehicle (AUV) performing surveys around the instrument, aided in determining conditions that affected the ESP mooring and provided context to the *in situ* processed samples.

The ESP was deployed during the spring-summer upwelling season of the Monterey Bay. The ESP detected changes in microbial abundances of the targeted genes that corresponded with different oceanographic regimes; marine crenarchaea were most abundant during the strong upwelling period and the Synechococcus bloomed late in the deployment when local advective processes dominated. SAR11 showed the least change; it was abundant throughout the deployment regardless of environmental conditions. The distributions of the bacterioplankon groups were similar to previously published distributions in Monterey Bay [6], [35], [44], [53], [54]. However, as previous studies of marine crenarchaeal population dynamics indicated its abundance at depth and seasonally occurring at the surface coinciding with upwelling conditions [50], [53], [55]–[57], the association of marine crenarchaea with high nitrate, low salinity water was unexpected. Low salinity intrusions originating offshore do enter the Monterey Bay [58], but associated nitrate concentrations are typically low. The results of this study have thus motivated investigation of how subsurface low-salinity intrusions may become nutrient-enriched, and what the causative processes mean for shelf nutrient budgets. Results from the field deployed ESP-MFB provide proof that the system and methods defined here were capable of detecting such well documented changes in gene abundance and show its potential in discovering alternative niches of specific bacterioplankton groups that were previously not known.

### Conclusions and future directions

In addition to bacterioplankton detection, the ESP has previously shown its utility in processing samples for harmful algae [15], [28], [30], [33], [34], [38], [45] and invertebrates [29], [32]; the addition of a qPCR capability creates new opportunities for utilizing this technology and provides an additional tool to augment autonomous ocean sensing networks. While our protocols are sufficient for migrating pre-existing qPCR assays from lab use to the ESP, there remain many opportunities for improving this procedure and experimenting with other polymerases, multiplexing strategies, alternative reagent storage, and the incorporation of template extraction controls, etc.

To our knowledge, this report is the first *in situ* oceanic application of qPCR in an autonomous field deployable instrument. We are working to extend that capability by combining it with new methods of sample archival [37] and event-triggered sample acquisition driven by sensors bundled with the ESP or available through a distributed sensor network. With the expansion of coastal and global ocean observatories, we anticipate new opportunities for developing and fielding ecogenomic sensors on fixed or mobile platforms [33], [36]. For the first time ever, ocean observing systems will allow investigators to carry out interactive, molecular analytical experiments remotely to test hypotheses and conduct routine monitoring. With the ESP now being available commercially (Spyglass, Marina, CA), we anticipate greater availability of the platform and wider spread usage in the near future.
